# Gender-Specific DNA Methylome Analysis of a Han Chinese Longevity Population

**DOI:** 10.1155/2014/396727

**Published:** 2014-04-14

**Authors:** Liang Sun, Jie Lin, Hongwu Du, Caiyou Hu, Zezhi Huang, Zeping Lv, Chenguang Zheng, Xiaohong Shi, Yan Zhang, Ze Yang

**Affiliations:** ^1^The Key Laboratory of Geriatrics, Beijing Hospital and Beijing Institute of Geriatrics, Ministry of Health, Beijing 100730, China; ^2^Key Laboratory of Nutrition and Metabolism, Institute for Nutritional Sciences, Shanghai Institutes for Biological Sciences, Chinese Academy of Sciences, University of Chinese Academy of Sciences, Shanghai 200031, China; ^3^Key Laboratory of Systems Biology, Shanghai Institutes for Biological Sciences, Chinese Academy of Sciences, University of Chinese Academy of Sciences, Shanghai 200031, China; ^4^University of Science and Technology Beijing, Beijing 100083, China; ^5^Department of Neurology, Jiangbin Hospital, Nanning, Guangxi 530021, China; ^6^Yongfu Committee of the Chinese People's Political Consultative Conference, Yongfu, Guangxi 541800, China; ^7^Department of Cardiothoracic Surgery, Guangxi Maternal and Child Health Hospital, Nanning, Guangxi 530003, China

## Abstract

Human longevity is always a biological hotspot and so much effort has been devoted to identifying genes and genetic variations associated with longer lives. Most of the demographic studies have highlighted that females have a longer life span than males. The reasons for this are not entirely clear. In this study, we carried out a pool-based, epigenome-wide investigation of DNA methylation profiles in male and female nonagenarians/centenarians using the Illumina 450 K Methylation Beadchip assays. Although no significant difference was detected for the average methylation levels of examined CpGs (or probes) between male and female samples, a significant number of differentially methylated probes (DMPs) were identified, which appeared to be enriched in certain chromosome regions and certain parts of genes. Further analysis of DMP-containing genes (named DMGs) revealed that almost all of them are solely hypermethylated or hypomethylated. Functional enrichment analysis of these DMGs indicated that DNA hypermethylation and hypomethylation may regulate genes involved in different biological processes, such as hormone regulation, neuron projection, and disease-related pathways. This is the first effort to explore the gender-based methylome difference in nonagenarians/centenarians, which may provide new insights into the complex mechanism of longevity gender gap of human beings.

## 1. Introduction

Over the last 100 years, humans experienced a huge increase of life expectancy. These advances were largely driven by extrinsic improvements of their living environment (such as diet and disease prevalence) as well as genetic variations (such as polymorphism and DNA methylation). Since human aging and longevity is a very complex trait where environmental, genetic, and stochastic factors are involved, it has largely aroused the attention of scientists around the world. A great number of studies have been carried out to investigate the mechanisms and key factors that may influence human mortality, aging, and lifespan [1–5].

As specific cohorts, nonagenarians and centenarians are always considered as the most valuable models to study the mechanisms involved in human aging and longevity [6]. They are considered to have reached the extreme limits of human life span but still show relatively good health conditions to maintain physiological function and escape the common fatal diseases [7]. Despite the increasing numbers of very old people worldwide, both nonagenarians and centenarians are still few from a demographic point of view. For example, in USA, nonagenarians now represent ~4.7% of the 65-and-older population whereas centenarians account for 17.3 per 100,000 people. Thus, it should be important to understand genetic and other factors, as well as the ways involved in healthy aging and longevity.

Currently, the majority of genome-based studies focused on the association between longevity and sequence variations including single nucleotide polymorphism or copy number variation [7–12]. In addition, epigenetic regulations, such as DNA methylation and histone modification, have emerged as a key driver of cell fate and their disruption could be related to a variety of human diseases [13–17]. Furthermore, with the development of genome-wide epigenetic analysis, much work has been carried out on epigenetic mechanisms of genome regulation during aging [18–20]. For example, heritable changes to the epigenome at both early and late life stages [21, 22], immune system/tissues specific variations [23–25], and dynamic epigenetic modifications through the lifespan [26, 27] have been reported to be responsible for many biological processes during healthy aging and longevity. Very recently, Heyn et al. found that the centenarian DNA had a genome-wide lower DNA methylation content and a reduced correlation in the methylation status of neighboring cytosine-phosphate-guanosine (CpG) sites in comparison with the newborn DNA [28]. This study demonstrated for the first time that the DNA methylomes at the two extremes of the human lifespan are distinct.

A significant trend observed in most parts of the world is that females have a longer life span than males. In particular, when nonagenarians and centenarians are considered, the male/female ratio has been reported to range between 1 : 4 and 1 : 7 [29]. Such a gender gap is quite remarkable, which has challenged scientists for decades to investigate possible reasons, such as better living conditions, specific biological advantages, and fewer behaviors that are bad for health compared to men [30–32]. A number of genome-based studies have been carried out to identify factors that may influence the gender difference based on animal models [33–35]. Recently, researchers have started to analyze gender-based genetic variations using human samples [36, 37]. It was suggested that the role of gender in the regulation of longevity may be linked to gender-specific genetic differences, such as the expression of sex hormone patterns and the changes in these patterns during lifetime [38]. However, so far it is difficult to collect enough samples to conduct a population-based longevity study. Moreover, gender-based DNA methylation analysis of the longevity population is not yet available, which may provide useful information with respect to epigenetic regulation of the longevity gender gap.

China has the largest population of adults aged 60+ years in the world [39]. In South China, there are several “longevity counties" due to the high number of nonagenarians and centenarians living there, such as Yongfu County, which has been qualified as the “Longevity Town” by Geriatric Society of China in 2007. In this study, a total of 200 Han nationality nonagenarian/centenarian participants (100 men and 100 women) from Yongfu County were recruited. We used a pool-based strategy to perform epigenome-wide investigation of DNA methylation profiles in male and female cohorts using the Illumina 450 K Methylation Beadchip. Differentially methylated CpGs between male and female samples and related genes were identified. To our knowledge, this is the first effort with such a large sample size of nonagenarians/centenarians to study the methylome difference that may contribute to the longevity gender gap.

## 2. Materials and Methods

### 2.1. Subjects

This project is an extension of the “Longevity and Health of Aging Population in Guangxi China” project conducted in 2008 and 2010 [40]. One hundred pairs of geography and nationality matched male and female volunteers aged 95+ years from urban and rural areas of Yongfu County, South China, were enrolled after exclusion of the subjects undertaking drug treatment. The male group (mean age 97.34 ± 2.66 years) was comprised of 94 nonagenarians (aged 95–99 years) and 6 centenarians (aged 100–105 years). The female group (mean age 99.14 ± 2.20 years) was comprised of 80 nonagenarians (aged 95–99 years) and 20 centenarians (aged 100–106 years). All subjects self-reported as Han nationality. The study was conducted according to the principles expressed in the Declaration of Helsinki. The Ethics Committee of Beijing Hospital, Ministry of Health, approved the study protocol. After the protocol was explained to the subjects, they provided written informed consent.

### 2.2. Genomic DNA Isolation and Pooling

Peripheral blood mononuclear cells (PBMCs) were isolated from whole blood for genomic DNA extraction using the Qiagen mini kit (Qiagen, Germany) following the manufacturer's protocol. DNA concentrations were determined by NanoDrop microvolume quantitation assay and 1% agarose electrophoresis. After validation of quality and integrity of individual genomic DNA, we equally pooled each sample into male and female groups, respectively.

### 2.3. Genome-Wide DNA Methylation Assay

The prepared genomic DNA (0.5 *μ*g) was bisulfate-converted with the EZ DNA Methylation Gold kit (Zymo Research, USA). After bisulfite conversion, each pooled sample was whole-genome amplified, enzymatically fragmented, precipitated, resuspended, and hybridized at 48°C for 16 h to Illumina Human Methylation 450 K BeadChip containing 485,577 locus-specific oligonucleotide primers. The probes were distributed among 20,216 transcripts, potential transcripts, or isolated CpG islands (CGIs). IlluminaHiScan SQ scanner was used for detection by fluorescent single-base primer extension assay. The methylation score is represented as *β*-value, a continuous parameter between 0 and 1 to show the ratio of the methylated-probe signal to total locus signal intensity. CpGs with a detection *P* value (representing the measured signal compared to negative controls) >0.05 were removed from the raw data. Raw data were further normalized using Illumina's control probe scaling procedure and background subtraction [41].

### 2.4. Differential Methylation Analysis

As described above, the measurement of whole genome DNA methylation used a pool-based approach, in which the *β*-value of each probe represents the average methylation level among all samples in the pool. To avoid sex-biased DNA methylation differences, we excluded methylation data for the* X* and* Y* chromosomes (473,864 probes remained). To identify differentially methylated probes (DMPs), we first assumed that the methylation levels for the whole genome obey a Gaussian model which could be used to predict DMPs [42]. Two simulation data sets which follow the Gaussian model with the same mean, standard variation, and sample size for male and female were randomly created, respectively. Then we calculated the different degrees of methylation changes between the two data sets (Δ_Me_, female-male). Since Δ_Me_ obeys the normal distribution, we built the normal distribution with the same mean and variance of Δ_Me_ and calculated the prediction intervals corresponding to a *P* value <0.05. After repeating this process 1000 times, the cutoff of 95% confidence interval was 0.197 ± 0.067. Therefore, a threshold of a 0.20 of Δ_Me_ was finally used to identify DMPs.

### 2.5. Bioinformatics Analysis

Gene ontology (GO) and KEGG (Kyoto encyclopedia of genes and genomes) pathway enrichment analyses were conducted using the *R* (version 2.14.0) package GOstats (version 2.28.0) [43]. GO terms and KEGG information were downloaded from Bioconductor (http://www.bioconductor.org). The *P* value was initially calculated based on hypergeometric distribution and filtered by adjusted *P* value <0.05. Multiple comparison adjustment was applied to get the adjusted *P* value using the false discovery rate (FDR) approach by *R* [44, 45].

## 3. Results and Discussion

It has been suggested that DNA pooling allows accurate assessment of average DNA methylation in large groups of individual genomes [46, 47]. Here, we applied this strategy to compare genome-wide methylation patterns between male and female groups of Han Chinese nonagenarians/centenarians.

### 3.1. General Analysis of DNA Methylomes of the Chinese Longevity Population

**Figure 1:**
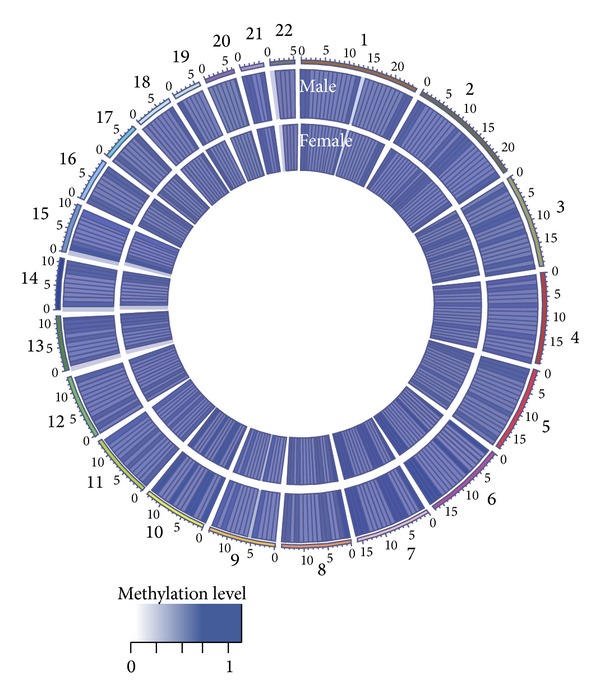
General view of DNA methylation level for autosomes of Chinese nonagenarians/centenarians. The average genome-wide DNA methylation levels in male and female samples are represented using Circos. The inner and outer tracks indicate the average methylation levels for female and male samples, respectively. All autosomes are represented via 10 Mbp-wide windows. The average methylation level in each region represents the average *β*-value (0-1) for all the probes in this region.

A general view of whole genome methylation profiles in autosomes of male and female nonagenarians/centenarians from Yongfu County in China was shown in Figure 1, using Circos software [48]. It appeared that the majority of the methylated regions have quite similar methylation patterns between male and female samples, implying that DNA methylation-based epigenetic profiles might be mostly common and gender-independent in the longevity population. Further analysis of the average methylation level of all examined CpGs confirmed that there is no significant difference between male samples (0.4962) and female ones (0.4974) at the whole genome level (using* t* test, *P* = 0.2452). However, a significant number of gender-specific DNA methylation differences between male and female samples were identified, which might play a role in gender-specific life span extension, for example, different gene expression regulation.

### 3.2. Identification of Differentially Methylated Probes and Related Genes

**Figure 2:**
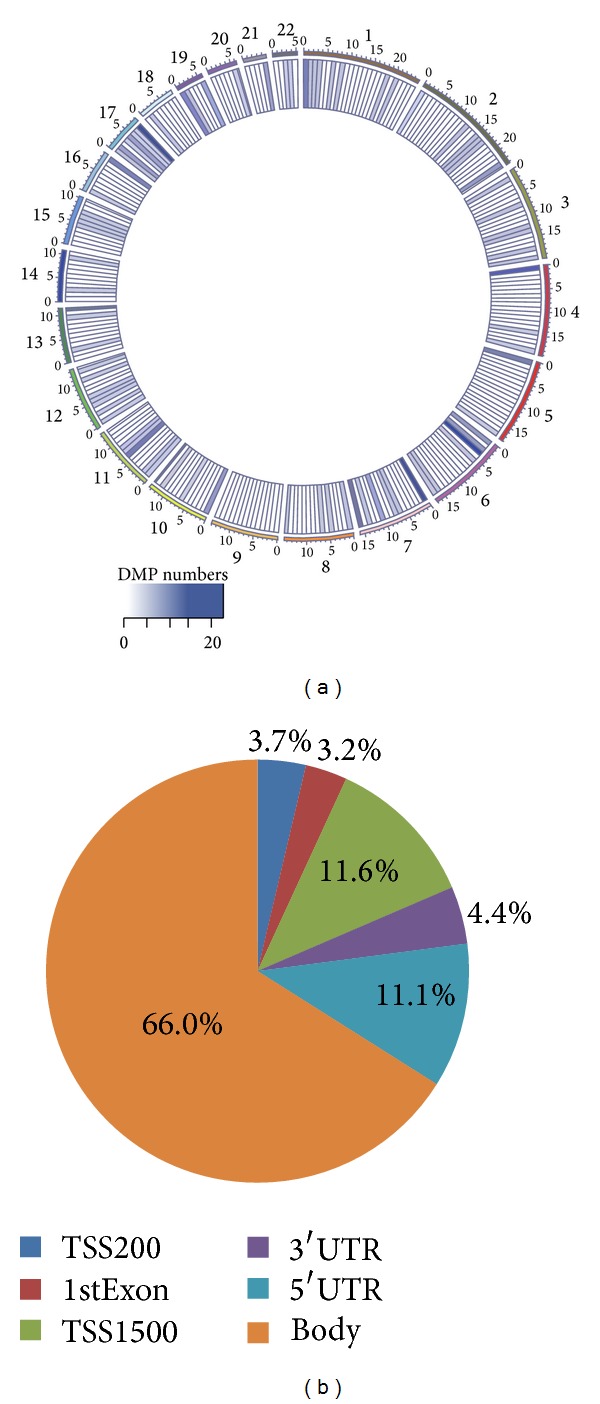
Distribution of DMPs between male and female samples. (a) Circos representation of the total number of DMPs in each region. The number is calculated using 10 Mbp-wide windows for each autosome. (b) Distribution of DMPs according to different regions of genes.

The discrepancies of DNA methylomes between male and female nonagenarians/centenarians prompted us to search for particular DMPs. In this study, DMPs were predicted based on a Gaussian model with the same mean and variation of Δ_Me_ (see Section 2). Using the male samples as control, hypermethylated and hypomethylated probes in female samples were selected if Δ_Me_ > 0.2 or < −0.2, respectively. Based on these criteria, we identified 850 DMPs (0.179% of all examined CpGs in autosomes), which are illustrated in Figure 2(a). These DMPs appeared to be enriched in certain genomic regions, especially in chromosome 17, which has been reported to contain many disease-associated genes [49]. This interesting finding implied that DMPs enriched in these chromosomal regions may play an important role in longevity gender gap.

We further examined the location of DMPs based on different parts of genes: 1500 bp above transcription start site (TSS1500), 200 bp above TSS (TSS200), 5′ untranslated region (5′-UTR), the 1st exon, gene body (other exons except the 1st exon), and 3′-UTR (Figure 2(b)). Most DMPs were enriched in gene body (66.0%). Although it has been reported that the methylation level of CpGs in coding region may regulate gene transcriptional activity [50], it is unclear whether DMPs detected in this study could affect the expression of corresponding genes. On the other hand, 15.4% DMPs were observed in the potential promoter regions (TSS1500 + TSS200) of genes. Thus, it is possible that some of these DMPs may be related to distinct expression difference of certain genes between men and women.

**Figure 3:**
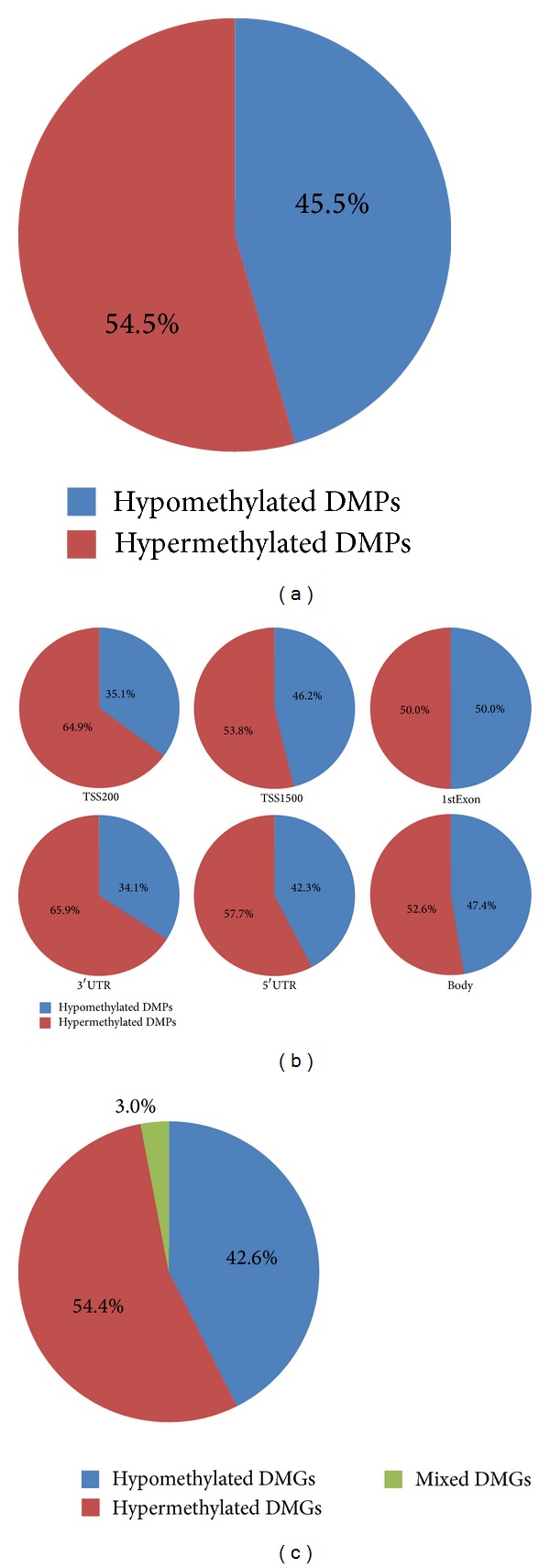
Distribution of hypermethylated and hypomethylated DMPs and DMGs. (a) Distribution of hypermethylated and hypomethylated DMPs. (b) Distribution of hypermethylated and hypomethylated DMPs in different parts of genes. (c) Distribution of hypermethylated, hypomethylated, and mixed DMGs.

We also analyzed the trend of methylation changes of DMPs. The majority of DMPs (54.5%) were hypermethylated in female compared to those in male samples (Figure 3(a)). Further analysis of different parts of genes revealed that, except the first exon, there were more hypermethylated DMPs than hypomethylated DMPs in all parts of genes (Figure 3(b)). These results implied that a more significant trend of DNA hypermethylation in females may be related to the gender gap in life expectancy.

To investigate the potential relationship between DMPs and genes, all DMPs were mapped to 564 genes (named differential methylated genes or DMGs; see Table S1 in Supplementary Material available online at http://dx.doi.org/10.1155/2014/396727). Here, a hypermethylated DMG was defined if it only contains hypermethylated DMPs. Similarly, a hypomethylated gene was defined if only hypomethylated DMPs were detected. If a gene contains both hypermethylated and hypomethylated DMPs, it was considered as a “mixed” DMG. In this study, 54.4%, 42.6%, and 3.0% of DMGs were found to belong to hypermethylated, hypomethylated, and mixed DMG groups. Thus, it appears that almost all DMGs have remained with a consistent trend of methylation changes.

It is known that epigenetic changes may affect the aging process and may be one of the central mechanisms of many age-related diseases [51]. In addition, it has also been reported that human disease genes are much closer to aging genes than expected by chance [52]. To investigate the potential relationship between DMGs detected in this study and aging or disease genes, we compared DMGs with known human aging genes and disease genes (provided in [52]), respectively. Few common genes could be found for both aging and disease genes (Tables S2 and S3), suggesting that the longevity gender gap might be unrelated to known aging or disease-related genes or processes. In other words, male and female longevities may share similar antiaging or antidisease mechanisms.

### 3.3. GO and KEGG Functional Enrichment Analysis of Differentially Methylated Genes

To extrapolate the biological processes of DMGs, a *R* package GOstats [43] was used to perform GO term and KEGG pathway enrichment analyses. Interestingly, no significant overlaps of GO terms could be found between hypomethylated and hypermethylated DMGs (Table 1).

Hypomethylated DMGs were mainly enriched in cellular component organization, cell surface receptor signaling, hormone regulation, and some disease-related pathways (such as Wnt receptor signaling pathway). It has been known for a long time that Wnt signaling pathway may lead to tumor development [53–55] and ROS-induced damage [56]. Some of the DMGs, such as chloride channel 7 (CLCN7), alpha-1 type I collagen (COL1A1), and estrogen receptor 1 (Esr1), are known to be associated with osteoporosis and fractures that are more common in women [57, 58]. Thus, hypomethylation of these genes may help extend the life span of women. In addition, some of these DMGs are involved in maintenance of cellular homeostasis (such as GO:0071840, GO:0071299, GO:0071842, and GO:0000904). It has been reported that a reduced homeostatic ability in response to internal or external stimuli may increase the occurrence of many diseases even death [59].

On the other hand, hypermethylated DMGs were found to be enriched in cell morphogenesis, cell-cell junction, and cell projection. Some of the enriched biological processes are known to be involved in neuron projection and central nervous system development [60]. In addition, the insulin-like growth factor 2 receptor (IGF2R) enriched in GO:0004714 (glycoprotein binding) could reduce the insulin resistance, which has been reported to increase life span more in females than in males [61, 62]. Our results suggested that DNA hypomethylation and hypermethylation may regulate corresponding genes involved in different processes. Therefore, identification of gender-specific methylation patterns may provide important information regarding the possible epigenetic mechanisms of longevity gender gap.

KEGG analysis showed that very few pathways could be significantly enriched for either hypomethylated or hypermethylated DMGs (Table 2). Hypomethylated DMGs were only enriched in extracellular matrix- (ECM-) receptor interaction (KEGG:04512), whereas hypermethylated DMGs were enriched in axon guidance (KEGG:04360) and cell adhesion molecules (CAMs) (KEGG:04514). The relationship between these pathways and gender-specific longevity is not clear.

### 3.4. Investigation of Hypomethylation Status of* X* Chromosome

Although the* X* chromosome contributes to the gender-specific methylation discrepancies, we could not analyze the complete methylation data for this chromosome because the “silent”* X* chromosome may cause bias when analyzing the DNA methylation level in women using our criteria. However, we could still identify its hypomethylated DMPs. In this study, we found 185 hypomethylated DMPs that correspond to 95 hypomethylated DMGs. Most of the hypomethylated DMPs were enriched in gene body (34.0%) and 5′-UTR (36.1%). Some DMGs either are known to be associated with hormonal effects (such as androgen receptor, AR) or have been considered as age-related genes in human cerebral cortex [63]. This observation suggested that* X*-linked hypomethylated DMGs may contribute to gender gap of human longevity, which is consistent with the hypothesis that gender-specific regulation of longevity may be related to the expression of sex hormone patterns [38]. Similar to autosomes, few common genes could be found while comparing the DMGs in* X* chromosome with known aging and disease genes (Tables S2 and S4).

### 3.5. Ongoing Work: Comparative DNA Methylome Analysis of Adults and Nonagenarians/Centenarians

We are collecting samples for examining the DNA methylomes of male and female adults from Yongfu County to identify epigenetic patterns that may be related to the mechanisms of longevity in the Han Chinese population. Based on our preliminary data, less DMPs in autosomes and more DMPs in sex chromosomes were observed in adults than in nonagenarians/centenarians (unpublished data). The average methylation level was significantly higher in both male and female adult samples compared to those in nonagenarians/centenarians, implying that hypomethylation in certain genomic regions may be related to longer life span. Hormone regulation and cell morphogenesis regulation appeared to be important for gender-specific longevity. These findings may help us figure out the difference of aging process between male and female. However, as longevity is a very complex trait, it should be understood that reliance on a single aspect of genetics has its limitations. In the future, by increasing the sample size, generating different levels of data, and developing more reliable methods, these defects may be rectified, providing scientists with more opportunities to explore in detail the role of DNA methylation and other factors involved in gender-specific longevity.

## 4. Conclusions

In summary, we are unique in reporting a comprehensive comparison of DNA methylome between male and female nonagenarians/centenarians in a Han Chinese population. The average methylation level in female samples was similar to that in male samples in spite of the fact that a significant number of DMPs were identified. These DMPs prefer to be enriched in certain chromosome regions. Further analysis of DMPs in different parts of genes revealed that most of them are located in gene body regions. Almost all of the DMGs are solely hypermethylated or hypomethylated. Functional enrichment analysis of these genes revealed that DNA hypermethylation and hypomethylation may regulate genes involved in different processes or pathways, some of which may contribute to the gender gap of life span. In addition, identification of* X*-based hypomethylated probes and genes could provide evidence for better understanding of the mechanism of longer lives in females.

## Supplementary Material

Table S1. DMGs detected in autosomes. Here, the methylation status represented the DMG types. A hypermethylated DMG (annotated as ‘+') was defined if it only contains hypermethylated DMPs. Similarly, a hypomethylated gene (described as ‘-'), was defined if only hypomethylated DMPs were detected. If a gene contains both hypermethylated and hypomethylated DMPs, it was considered as a “mixed” DMG which was noted as ‘mixed'.
Table S2. Overlaps between DMGs and known human aging genes. This table shows the list of DMGs which overlapped with known aging genes in both autosomal and X chromosomes. The status showed the type of DMGs.
Table S3. Overlaps between autosomal DMGs and known disease genes. This table shows the overlaps between autosomal DMGs and known disease genes.
Table S4. Overlaps between DMGs in X chromosome and known disease genes. This table shows the Overlaps between DMGs in X chromosome and known disease genes.

## Figures and Tables

**Table 1:** GO analysis of hypomethylated and hypermethylated DMGs.

GO ID	Description	*P* value	FDR
Hypomethylated genes			
Biological process			
GO:0016043	Cellular component organization	5.03*E* − 07	1.06*E* − 03
GO:0071840	Cellular response to vitamin A	1.74*E* − 06	1.83*E* − 03
GO:0071299	Cell surface receptor linked signaling pathway	2.27*E* − 04	2.36*E* − 02
GO:0007166	Regulation of hormone levels	3.95*E* − 04	1.36*E* − 02
GO:0010817	Cell projection organization	4.08*E* − 04	2.36*E* − 02
GO:0030030	Hormone secretion	4.96*E* − 04	2.36*E* − 02
GO:0046879	Cellular component organization at cellular level	6.80*E* − 04	4.36*E* − 02
GO:0071842	Cellular response to vitamin	6.81*E* − 04	4.36*E* − 02
GO:0071295	Regulation of transcription from RNA polymerase II promoter	7.23*E* − 04	4.36*E* − 02
GO:0006357	Epithelial cell development	8.35*E* − 04	4.59*E* − 02
GO:0002064	Hormone transport	8.52*E* − 04	4.59*E* − 02
GO:0009914	Production of molecular mediator involved in inflammatory response	8.54*E* − 04	4.59*E* − 02
GO:0002532	Cell morphogenesis involved in differentiation	8.77*E* − 04	4.59*E* − 02
GO:0000904	Transmembrane receptor protein tyrosine kinase signaling pathway	9.59*E* − 04	4.94*E* − 02
GO:0007169	Wnt receptor signaling pathway	9.71*E* − 04	4.94*E* − 02
Cellular component			
GO:0015629	Actin cytoskeleton	1.94*E* − 03	4.99*E* − 02

Hypermethylated genes			
Biological process			
GO:0000902	Cell morphogenesis	3.23*E* − 05	3.54*E* − 02
GO:0021955	Central nervous system neuron axonogenesis	3.39*E* − 05	3.54*E* − 02
GO:0048869	cellular developmental process	4.21*E* − 05	3.54*E* − 02
GO:0032989	cellular component morphogenesis	7.81*E* − 05	3.73*E* − 02
GO:0048858	cell projection morphogenesis	8.50*E* − 05	3.73*E* − 02
GO:0032990	cell part morphogenesis	1.01*E* − 04	3.73*E* − 02
GO:0051179	localization	1.04*E* − 04	3.73*E* − 02
GO:0048667	cell morphogenesis involved in neuron differentiation	1.26*E* − 04	3.96*E* − 02
GO:0030154	cell differentiation	1.71*E* − 04	4.80*E* − 02
Cellular component			
GO:0044459	plasma membrane part	1.05*E* − 05	1.99*E* − 03
GO:0016020	membrane	3.19*E* − 05	1.99*E* − 03
GO:0044425	membrane part	4.55*E* − 05	1.99*E* − 03
GO:0005911	cell-cell junction	4.67*E* − 05	2.16*E* − 03
GO:0030054	cell junction	6.34*E* − 05	4.65*E* − 03
Molecular function			
GO:0015108	chloride transmembrane transporter activity	3.46*E* − 05	1.99*E* − 03
GO:0015103	inorganic anion transmembrane transporter activity	6.03*E* − 05	2.16*E* − 03
GO:0015296	anion:cationsymporter activity	1.17*E* − 04	2.65*E* − 02
GO:0004714	glycoprotein binding	1.71*E* − 04	4.80*E* − 02

**Table 2:** KEGG pathway enrichment analysis of hypomethylated and hypermethylated DMGs.

KEGG ID	Description	*P* value	FDR
Hypomethylated genes			
KEGG:04512	ECM-receptor interaction	2.50*E* − 02	7.50*E* − 03
Hypermethylated genes			
KEGG:04360	Axon guidance	3.7*E* − 3	1.48*E* − 02
KEGG:04514	Cell adhesion molecules (CAMs)	1.6*E* − 2	3.20*E* − 02
